# Prevalence and risk factors for stunting and severe stunting among under-fives in North Maluku province of Indonesia

**DOI:** 10.1186/1471-2431-9-64

**Published:** 2009-10-06

**Authors:** Ramli, Kingsley E Agho, Kerry J Inder, Steven J Bowe, Jennifer Jacobs, Michael J Dibley

**Affiliations:** 1 Centre for Clinical Epidemiology and Biostatistics, the University of Newcastle, NSW, Australia; 2 School of Medicine, the University of Western Sydney, NSW, Australia; 3 School of Public Health, the University of Sydney, NSW, Australia

## Abstract

**Background:**

Adequate nutrition is needed to ensure optimum growth and development of infants and young children. Understanding of the risk factors for stunting and severe stunting among children aged less than five years in North Maluku province is important to guide Indonesian government public health planners to develop nutrition programs and interventions in a post conflict area. The purpose of the current study was to assess the prevalence of and the risk factors associated with stunting and severe stunting among children aged less than five years in North Maluku province of Indonesia.

**Methods:**

The health and nutritional status of children aged less than five years was assessed in North Maluku province of Indonesia in 2004 using a cross-sectional multi-stage survey conducted on 750 households from each of the four island groups in North Maluku province. A total of 2168 children aged 0-59 months were used in the analysis.

**Results:**

Prevalence of stunting and severe stunting were 29% (95%CI: 26.0-32.2) and 14.1% (95%CI: 11.7-17.0) for children aged 0-23 months and 38.4% (95%CI: 35.9-41.0) and 18.4% (95%CI: 16.1-20.9) for children aged 0-59 months, respectively. After controlling for potential confounders, multivariate analysis revealed that the risk factors for stunted children were child's age in months, male sex and number of family meals per day (≤2 times), for children aged 0-23 months, and income (poorest and middle-class family), child's age in months and male sex for children aged 0-59 months. The risk factors for severe stunting in children aged 0-23 months were income (poorest family), male sex and child's age in months and for children aged 0-59 months were income (poorest family), father's occupation (not working), male sex and child's age in months.

**Conclusion:**

Programmes aimed at improving stunting in North Maluku province of Indonesia should focus on children under two years of age, of male sex and from families of low socioeconomic status.

## Background

The optimal growth and development of infants and young children are fundamental for their future [1]. Stunting, a deficit in height or length relative to a child's age is a major health problem in South Asia where half of children aged less than five years are stunted [2]. In Indonesia, 37% of children aged less than five years are stunted [3]. Promoting better eating habits in an effort to improve nutrition is one of the most challenging tasks in Indonesia as malnutrition remains one of the most important public health problems facing almost every district [4].

In Indonesia, like many developing countries, the most common nutritional problems in infancy and early childhood are stunting, wasting; iron-deficiency anaemia, poverty and low birth weight [5,6]. Malnutrition during the first 2 years of life can lead to mortality and morbidity in childhood [7,8] and is one of the most preventable risk factors for mortality [9].

Past studies have also shown that lower intelligence quotient (IQ), mother's height, male sex, mother and father level of education, poverty, socioeconomic status, residence, child care behaviour (inadequate complimentary feeding and breastfeeding), cultural beliefs, access to health care and environmental ecosystems [10,11] are factors associated with stunting in children aged less than five years.

Despite the persistently high prevalence of stunted children in Indonesia, there is a lack of information about the prevalence and risk factors associated with stunted and severely stunted children in the North Maluku province of Indonesia using the new Growth reference from the World Health Organisation [12]. This province is an area in Indonesia that in 2004 had recently emerged from a period of prolonged civil conflict. This paper assesses the prevalence and risk factors associated with stunting and severe stunting in children aged 0-59 months old in North Maluku province of Indonesia.

## Methods

### Study location

**Figure 1:**
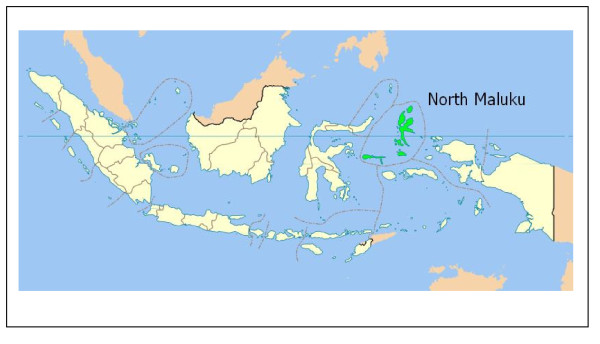
**Map of Indonesia showing North Maluku Province**. (Source: Wikipedia, 2008)

The study covered all areas in the North Maluku province of Indonesia (see Figure 1) [13] with a total population of about 920,000 people in 2006 [14,15], divided into four island groups The first island group consists of the districts of Ternate and Tidore with a total population of about 241,000 people. The second island group consists of the districts of Central Halmahera and East Halmahera with a total population of about 95,000 people. The third island group consists of the districts of West Halmahera and North Halmahera with a total population of about 276,000 people. The forth island group consists of the districts of South Halmahera and Sula-Isles with a total population of about 308,000 people [15].

### Study design

A cross-sectional survey was conducted in 2004 on 3000 households from the four island groups in North Maluku province. A multistage cluster sampling technique was used for selecting the study sample in which North Maluku province was grouped into four island groups with eight districts in total.

### Selection of subjects

The four island groups within North Maluku province were used to select the study areas. In the first stage, two districts were randomly selected based on probability proportional to size from each island group [15]. In the second stage, subdistricts (referred to as clusters) were randomly selected from each district. In the third stage, Puskesmas (Community Public Health Services) were selected randomly from each subdistrict and finally, the villages were randomly selected from Puskesmas. Household selection in each cluster was randomly taken by using the sampling frame of every 10^th ^household with the nearest household from the village health service (Pustu) as the starting point. Comprehensive details of the study districts and selection criteria have been reported elsewhere [16]. In total, 50 households were selected in each cluster and 15 clusters in each island group, yielding a total of 750 households from each island group.

### Stunting (Height-for-age)

The nutritional status of children less than five years of age was measured anthropometrically. Length was measured for children aged less than two years old and height for those two years of age and older. Length was measured using a wooden stadiometer to the nearest 0.1 cm and height was measured using Microtoice tape to the nearest 0.1 cm [17]. The height-for-age measurement status was expressed in Standard Deviation (SD) units (Z-score) from the median of the reference population. Children with a measurement of <-2 SD units from the median of the reference population were considered short for their age (stunted) and children with measurement of <-3SD units from the median of the reference population were considered to be severely stunted.

### Socioeconomic factors

A structured household questionnaire was used for collecting information about the following family level factors: region (urban and rural), district (total of eight), father's level of education (completed elementary school [6 years of schooling], completed middle school [9 years of schooling] and completed high school [12 years of schooling]; mother's level of education; parental education (both with higher education, father with high education, mother with high education, neither with high education); father's occupation, mother's occupation; parental occupation; household wealth index (calculated from household's ownership of consumer items including refrigerators, VCR players, satellite dishes, televisions, lounges, boats, cars, motorcycles; flooring material; type of drinking-water source; toilet facilities; and other characteristics that are related to wealth status - categorised into poorest, middle and least poor); number of household members in the family and number of family meals per day. In addition, the following child level factors were collected: child's age in months; gender; provision of nutritional status information during pregnancy and number of antenatal visits. The questionnaires were administered after a signed informed consent was collected. The questionnaires were checked daily for accuracy, consistency, and completeness by field supervisors.

### Ethical permission

This study had the approval of the Ministry of Health in Indonesia and the protocol for secondary data analysis was approved by Human Ethics Research Committee, University of Newcastle - Australia.

### Statistical analyses

Data were entered into a computerised database and cleaned using the data entry program EPIINFO [18]. Data on nutritional status were analysed based on the new growth reference from the World Health Organisation. The household ownership of consumer items described above were used in constructing the wealth index scores by using a method similar to that described by Filmer and Pritchett [19] and were divided into three categories. The bottom 40% of the households was referred to as the poorest households, the next 40% as the middle-class households, and the top 20% as the least poor households. The analysis for stunted and severely stunted children was categorised into two groups (1) children aged 0-23 months, and (2) children aged 0-59 months.

To determine the level of stunting and severe stunting the dependent variable was expressed as a dichotomous variable: category 0 if not stunted (≥-2SD) or severely stunted (≥-3SD) and category 1 if stunted (<-2SD) or severely stunted (<-3SD).

Firstly, univariate binary logistic regression analysis was performed to examine the association between stunted and severely stunted children aged 0-23 months and stunted children 0-59 months. Secondly, the factors associated with stunting and severe stunting were examined in a multiple logistic regression model. A stepwise backward elimination approach was applied. At the start variables were selected for inclusion in the model if their univariate analysis p-value was <= 0.25. Only variables which were statistically associated with stunted and severely stunted children (p < 0.05) remained in the final model. Unadjusted and adjusted odds ratios from a logistic model are presented with 95% confidence intervals. The 'SVY' commands from Stata version 9.2 (Stata Corp) were used for data analysis to adjust for the cluster sampling design and appropriate sampling weights.

## Results

### Univariate Analyses

Table 1 presents the prevalence of stunting and severe stunting in children aged 0-23 months and 0-59 months, respectively. Table 1 reveals that the child's age and gender were significantly associated with stunting in children aged 0-23 months while the mother's education, the household wealth index, the child's age and gender were significantly associated with severe stunting in children aged 0-23 months.

**Table 1:** Prevalence of stunting and severe stunting in children aged 0-23 months and 0-59 months

**Characteristic**	**n**	**Stunted children 0-23 Months**	**Severely stunted children 0-23 Months**	**Stunted children 0-59 Months**	**Severely stunted children 0-59 Months**
		
		**% (95% CI)**	**% (95% CI)**	**% (95% CI)**	**% (95% CI)**
**Region**					
Rural	536	27.1 (21.0-34.3)	13.8 (9.5-19.5)	33.4 (28.6-38.6)*	15.9 (12.4-20.1)
Urban	1632	29.6 (26.2-33.2)	14.2 (11.4-17.6)	40.0 (37.2-42.9)	19.2 (16.4-22.3)
**District**					
Ternate	387	30.9 (23.8-39.1)	14.2 (9.9-19.9)	36.4 (31.0-42.2)	16.0 (12.3-20.6)
Tidore	149	19.1 (10.4-32.5)	12.8 (4.9-29.2)	25.5 (18.9-33.4)	15.4 (8.6-26)
Central Halmahera	280	32.3 (25.7-39.7)	15.5 (11.3-21)	42.9 (36.5-49.5)	23.2 (18.1-29.3)
East Halmahera	280	37.0 (28.3-46.7)	21.9 (13.2-34.3)	42.5 (33.2-52.4)	23.6 (14.6-35.8)
West Halmahera	178	23.7 (15.9-33.8)	6.2 (3.1-12.0)	38.2 (33.2-43.5)	16.3 (12.5-21)
North Halmahera	279	31.1 (24.5-38.6)	13.3 (9.1-19.1)	41.6 (34.5-49.0)	17.2 (12.6-23.0)
South Halmahera	446	24.7 (19.7-30.5)	12.4 (8.2-18.2)	38.6 (35.2-42.1)	19.3 (15.1-24.3)
Sula-Isles	169	27.4 (20.7-35.3)	10.7 (6.7-16.8)	34.3 (29.6-39.4)	11.2 (9-14.0)
**Household factors**					
**Father's education**					
Completed Elementary School (aged 7-12)	832	32.0 (27.2-37.2)	16.2 (12.6-20.7)	41.8 (37.8-45.9)*	20.9 (17.6-24.7)*
Completed Middle School (aged 13-15)	557	28.4 (23.4-34.0)	13.2 (9.7-17.6)	39.3 (35.2-43.6)	19.0 (15.5-23.2)
Completed High School (aged 16-18)	779	26.3 (22.0-31.2)	12.6(9.2-17)	34.0 (30.3-37.9)	15.2 (12.1-18.8)
**Mother's education**					
Completed Elementary School (aged 7-12)	1163	30.8 (27.0-34.9)	16.7 (13.6-20.30*	41.6 (38.4-44.9)**	20.9 (18.0-24.3)**
Completed Middle School (aged 13-15)	514	29.7 (24.5-35.5)	11.5 (8-16.4)	37.7 (32.8-43)	15.8 (12.4-19.8)
Completed High School (aged 16-18)	491	24.5 (19.6-30.2)	11.2 (7.7-16.1)	31.4 (27.0-36.0)	14.9 (11.7-18.8)
**Parental Education**					
Both with high education	871	26.2 (22.1-30.7)	11.3 (8.4-14.9)	33.6 (30-37.5)**	15.4 (12.7-18.6)*
Father with high education	134	33.3 (22.7-46.0)	12.0 (5.6-23.7)	41.0 (32.6-50.1)	14.9 (9.4-22.9)
Mother with high education	465	29.2 (23.5-35.7)	16.1 (11.8-21.5)	41.1 (37.1-45.1)	19.4 (15.6-23.8)
Neither with high education	698	31.7 (26.9-37.1)	17.1 (13.1-21.9)	41.9 (37.7-46.4)	22.1 (18.4-26.2)
**Provided with nutritional information during pregnancy (n = 2110)**					
No	729	29.9 (25.4-35.0)	16.0 (12.8-19.9)	42.0 (37.7-46.4)	21.1 (17.6-25.1)
Yes	1381	28.2 (24.9-31.9)	12.9 (10.1-16.3)	36.6 (33.7-39.7)	17.0 (14.3-20.1)
**Father's occupation**					
Any Labour	1396	29.3 (25.6-33.3)	15.1 (12.2-18.7)	40.0 (36.9-43.2)	19.3 (16.7-22.3)**
Fisher man	355	27.9 (22.6-33.8)	12.5 (8.5-18)	35.5 (30.8-40.5)	18.0 (14.5-22.1)
No work	60	35.9 (21.7-53.1)	20.5 (10.4-36,5)	45.0 (34.0-56.5)	28.3 (18.5-40.5)
Government private officer	357	27.7 (20.9-35.7)	10.8 (6.9-16.5)	33.9 (29-39.2)	13.2 (9.8-17.5)
**Mother's occupation**					
Any Labour	694	29.9 (25.3-34.9)	14.4 (11.4-18.2)	39.5 (35.4-43.8)	18.4 (15.4-22)
Fisher woman	96	30.6 (20.6-42.8)	18.4 (9.8-31.8)	38.5 (30.8-46.9)	22.9 (15.5-32.5)
No work	1302	28.4 (24.4-32.7)	13.6(10.4-17.6)	37.9 (34.5-41.4)	18.1 (15.2-21.5)
Government private officer	76	29.2 (17.7-44.0)	14.6 (8.0-25.0)	36.8 (27.6-47.2)	15.8 (10.2-23.7)
**Parental employment**					
Both working	838	29.8 (25.8-34.2)	14.9 (11.9-18.6)	38.7 (35.3-42.2)	18.1 (15.4-21.3)
Father only working	1270	28.1 (24.1-32.4)	13.2 (10.0-17.2)	37.9 (34.5-41.4)	18.0 (15.1-21.4)
Mother only working	28	33.3 (14.2-60.2)	13.3 (2.8-44.8)	53.6 (38.5-68.1)	35.7 (19.7-56.8)
Neither working	32	37.5 (21.1-57.4)	25.0 (11.7-45.7)	37.5 (23.1-54.6)	21.9 (11.4-38.0)
**Household wealth Index**					
Poorest	867	32.9 (27.6-38.7)	17.4 (13.4-22.4)**	43.1 (39.0-44.0)**	21.1 (17.4-25.4)**
Middle	867	28.2 (24.3-32.5)	13.9 (11.0-17.6)	37.4 (33.9-40.9)	18.9 (16.3-21.9)
Least Poor	434	23.4 (18.1-29.7)	8.3 (5.1-13.4)	30.9 (26.4-35.8)	11.8 (8.7-15.7)
**Household member**					
≤5 members	1359	31.3 (27.6-35.4)	14.8 (11.6-18.7)	39.3 (36.2-42.5)	18.5 (15.7-21.7)
6-12 members	809	24.9 (21-29.4)	12.9 (10.1-16.4)	36.8 (33.0-40.8)	18.1 (15.2-21.3)
**Number of family Meals per day**					
2 Times	675	24.9 (20.5-29.8)	13.1 (9.9-17.1)	36.3 (32.2-40.6)	17.6 (14.3-21.6)
>2 Times	1493	30.8 (27.0-34.8)	14.5 (11.5-18.2)	39.3 (36.22-42.5)	18.7 (16-21.8)
**Child level factors**					
**Child's age in category**					
0-5	266	12.8 (9.1-17.7)***	7.9 (5.6-11.0)***	12.8 (9.1-17.7)***	7.9 (5.6-11.0)***
6-11	365	24.1 (19.3-29.7)	10.1 (5.6-11.0)***	24.1 (19.3-29.7)	10.1 (6.6-15.2)
12-17	318	33.9 (28.6-39.7)	15.7 (5.6-11.0)***	33.9 (28.6-39.7)	15.7 (11.7-20.8)
18-23	234	48.3 (40.3-56.3)	25.2 (5.6-11.0)***	48.3 (40.3-56.3)	25.2 (18.8-32.9)
24-29	238			48.7 (42.2-55.4)	26.5 (20.4-33.6)
30-35	169			51.5 (42.9-60.0)	23.1 (16.8-30.8)
36-41	215			52.6 (45.9-59.1)	25.6 (20.5-31.5)
42-47	147			47.6 (37.7-57.7)	19.7 (13-28.8)
48-53	135			49.6 (39.9-59.4)	23.7 (16.8-32.3)
54-59	81			44.4 (34.6-54.8)	16.1 (8.7-27.6)
**Gender**					
Male	1115	32.2 (28.0-36.7)*	16.7 (13.2-21.1)**	41.4 (37.9-44.8)*	20.5 (17.4-24.1)**
Female	1053	25.6 (21.6-30.1)	11.3 (8.9-14.3)	35.2 (31.8-38.8)	16.1 (13.7-18.7)
**Antenatal visit (n = 2110)**					
No	91	39.6 (27.2-53.4)	16.7 (7.6-32.7)	44.0 (35.1-53.2)	23.1 (15.4-33.1)
Yes	2019	28.4 (25.4-31.5)	16.7 (11.5-16.6)	38.2 (35.6-40.9)	18.2 (15.9-20.8)

**Overall**		29.0 (26.0-32.2)	16.7 (11.7-17.0)	38.4 (35.9-41.0)	18.4 (16.1-20.9)

*P < 0.05; **P < 0.01, ***P < 0.001; X^2^-test was applied to test statistical significance

Weighted total was 2168 otherwise stated within brackets

For children aged 0-59 months, parental education (only mothers with higher education and neither parent with higher education), household wealth index (poorest), region (urban), child's age and males were statistically significantly associated with stunting. While, father's occupation (not working), parental education (neither parent with higher education), household wealth index (poorest), child's age (24-29 months old) and males reported higher prevalence of severe stunting. The overall prevalence of being stunted and severely stunted was 29% and 14.1% for children aged 0-23 months and 38.4% and 18.4% for children aged 0-59 months (see Table 1), respectively.

### Multivariate Analyses

Tables 2 and 3 shows the unadjusted and adjusted odds ratios for the association between stunted and severely stunted children and socioeconomic characteristics of children aged 0-23 months children aged 0-59 months.

**Table 2:** Risk factors for stunting in children aged 0-23 months and 0-59 months

**Characteristic**	**Stunted children 0-23 Months**		**Stunted children 0-59 Months**	
	
	**Unadjusted OR (95% CI)**	**Adjusted OR (AOR) (95% CI)**	**Unadjusted OR (95% CI)**	**AOR (95% CI)**
**Region**				
Rural	1.00		1.00	
Urban	1.13 (0.77-1.65)		1.33 (1.03-1.71)	
**District**				
Ternate	1.00		1.00	
Tidore	0.53 (0.24-1.17)		0.60 (0.38-0.94)	
Central Halmahera	1.06 (0.66-1.72)		1.31 (0.91-1.88)	
East Halmahera	1.31 (0.76-2.24)		1.29 (0.81-2.06)	
West Halmahera	0.69 (0.38-1.28)		1.08 (0.78-1.50)	
North Halmahera	1.01 (0.62-1.64)		1.24 (0.84-1.83)	
South Halmahera	0.73 (0.46-1.16)		1.10 (0.82-1.45)	
Sula-Isles	0.84 (0.50-1.41)		0.91 (0.66-1.26)	
**Household factors Father's education**				
Completed Elementary School (aged 7-12)	1.00		1.00	
Completed Middle School (aged 13-15)	0.84 (0.59-1.21)		0.90 (0.71-1.14)	
Completed High School (aged 16-18)	0.76 (0.55-1.05)		0.70 (0.57-0.91)	
**Mother's Education**				
Completed Elementary School (aged 7-12)	1.00		1.00	
Completed Middle School (aged 13-15)	0.95 (0.69-1.31)		0.85 (0.66-1.09)	
Completed High School (aged 16-18)	0.73 (0.55-0.97)		0.64 (0.50-0.83)	
**Parental Education**				
Both with high education	1.00		1.00	
Father with high education	1.41 (0.78-2.54)		1.37 (0.92-2.04)	
Mother with high education	1.17 (0.83-1.64)		1.38 (1.09-1.73)	
Neither with high education	1.31 (0.96-1.80)		1.43 (1.11-1.84)	
**Provided with nutritional information during pregnancy**				
No	1.00		1.00	
Yes	0.78 (0.56-1.07)		0.77 (0.57-1.03)	
**Father's occupation**				
Any Labour	1.00		1.00	
Fisher man	0.93 (0.66-1.32)		0.83 (0.65-1.06)	
No work	1.35 (0.65-2.80)		1.23 (0.77-1.96)	
Government private officer	0.92 (0.62-1.38)		0.77 (0.60-0.99)	
**Mother's occupation**				
Any Labour	1.00		1.00	
Fisherwoman	1.03 (0.58-1.83)		0.96 (0.65-1.42)	
No work	0.93 (0.69-1.25)		0.93 (0.75-1.17)	
Government private officer	0.96 (0.49-1.92)		0.89 (0.55-1.46)	
**Parental employment**				
Both working	1.00		1.00	
Father only working	0.92 (0.70-1.21)		0.97 (0.79-1.18)	
Mother only working	1.18 (0.39-3.59)		1.83 (0.97-3.47)	
Neither working	1.41 (0.62-3.20)		0.95 (0.47-1.92)	
**Household wealth Index**				
Poorest	1.00		1.00	1.00
Middle	0.80 (0.58-1.10)		0.87 (0.65-1.16)	0.78 (0.63-0.98)
Least Poor	0.62 (0.41-0.94)		0.50 (0.33-0.75)	0.62 (0.45-0.85)
**Household member**				
≤5 members	1.00		1.00	
6-12 members	0.73 (0.56-0.95)		0.90 (0.73-1.11)	
**Family Meals per day**				
2 Times	1.00	1.00	1.00	
>2 Times	0.74 (0.54-1.02)	0.70 (0.50-0.99)	0.88 (0.70-1.11)	
**Child level factors**				
**Child's age (months)**	1.10 (1.07-1.14)	1.11 (1.08-1.14)	1.03 (1.02-1.04)	1.03 (1.02-1.04)
**Gender**				
Male	1.00	1.00	1.00	1.00
Female	0.73 (0.54-0.97)	0.67 (0.50-0.89)	0.77 (0.63-0.95)	0.74 (0.59-0.93)
**Antenatal visit**				
No	1.00		1.00	
Yes	0.89 (0.60-1.32)		0.77 (0.57-1.03)	

**Table 3:** Risk factors for severe stunting in children aged 0-23 months and 0-59 months

**Characteristic**	**Severely stunted children 0-23 Months**		**Severely stunted children 0-59 Months**	
	
	**Unadjusted OR (95% CI)**	**Adjusted OR (95% CI)**	**Unadjusted OR (95% CI)**	**Adjusted OR (95% CI)**
**Region**				
Rural	1.00		1.00	
Urban	1.04 (0.64-1.70)		1.33 (1.03-1.71)	
**District**				
Ternate	1.00		1.00	
Tidore	0.88 (0.29-2.68)		0.60 (0.38-0.94)	
Central Halmahera	1.10 (0.64-1.92)		1.31 (0.91-1.88)	
East Halmahera	1.70 (0.81-3.56)		1.29 (0.81-2.06)	
West Halmahera	0.40 (0.18-0.92)		1.08 (0.78-1.50)	
North Halmahera	0.93 (0.51-1.68)		1.24 (0.84-1.83)	
South Halmahera	0.85 (0.46-1.57)		1.10 (0.82-1.45)	
Sula-Isles	0.72 (0.37-1.40)		0.91 (0.66-1.26)	
**Household factors Father's education**				
Completed Elementary School (aged 7-12)	1.00		1.00	
Completed Middle School (aged 13-15)	0.78 (0.48-1.26)		0.89 (0.64-1.24)	
Completed High School (aged 16-18)	0.75 (0.51-1.10)		0.68 (0.50-0.91)	
**Mother's education**				
Completed Elementary School (aged 7-12)	1.00		1.00	
Completed Middle School (aged 13-15)	0.65 (0.41-1.05)		0.70 (0.51-0.98)	
Completed High School (aged 16-18)	0.63 (0.43-0.92)		0.66 (0.49-0.88)	
**Parental education**				
Both with high education	1.00		1.00	
Father with high education	1.07 (0.44-2.63)		1.37 (0.92-2.04)	
Mother with high education	1.51 (0.97-2.35)		1.38 (1.09-1.73)	
Neither with high education	1.62 (1.09-2.41)		1.43 (1.11-1.84)	
**Provided with nutritional information during pregnancy**				
No	1.00		1.00	
Yes	0.78 (0.56-1.07)		0.77 (0.57-1.03)	
**Father's occupation**				
Any Labour	1.00		1.00	1.00
Fisherman	0.80 (0.49-1.32)		0.92 (0.70-1.19)	1.13 (0.85-1.52)
No work	1.45 (0.63-3.34)		1.65 (0.92-2.94)	2.04 (1.17-3.53)
Government private officer	0.68 (0.42-1.10)		0.63 (0.45-0.89)	0.79 (0.55-1.14)
**Mother's occupation**				
Any Labour	1.00		1.00	
Fisherwoman	1.33 (0.62-2.88)		1.31 (0.78-2.22)	
No work	0.93 (0.63-1.39)		0.98 (0.75-1.28)	
Government private officer	1.01 (0.51-2.01)		0.83 (0.49-1.40)	
**Parental employment**				
Both working	1.00		1.00	
Father only working	0.87 (0.60-1.26)		0.97 (0.79-1.18)	
Mother only working	0.88 (0.16-4.74)		1.83 (0.97-3.47)	
Neither working	1.90 (0.72-5.01)		0.95 (0.47-1.92)	
**Household wealth Index**				
Poorest	1.00	1.00	1.00	1.00
Middle	0.77 (0.51-1.15)	0.78 (0.52-1.18)	0.87 (0.65-1.16)	0.89 (0.66-1.20)
Least Poor	0.43 (0.24 -0.79)	0.42 (0.23-0.79)	0.50 (0.33-0.75)	0.52 (0.33-0.82)
**Household member**				
≤5 members	1.00		1.00	
6-12 members	0.86 (0.59-1.24)		0.90 (0.73-1.11)	
**Family Meals per day**				
2 Times	1.00		1.00	
>2 Times	0.89 (0.60-1.32)		0.93 (0.69-1.26)	
**Child level factors**				
**Child's age (months)**	1.08 (1.05-1.12)	1.08 (1.05-1.12)	1.03 (1.02-1.04)	1.02 (1.01-1.03)
**Gender**				
Male	1.00	1.00	1.00	1.00
Female	0.63 (0.45-0.89)	0.58 (0.42-0.81)	0.77 (0.63-0.95)	0.72 (0.58-0.90)
**Antenatal visit**				
No	1.00		1.00	
Yes	0.89 (0.60-1.32)		0.77 (0.57-1.03)	

#### Risk factors for stunting

The odds for stunted children aged 0-23 months was 26 percent lower in families that provided at least three meals per day. Increased child age in months was statistically associated with stunting in children aged 0-23 months (adjusted OR (AOR) = 1.11, 95%CI: 1.08 - 1.14; p < 0.001) and girls had reduced odds of being stunted compared to boys (AOR = 0.67, 95%CI: 0.50 - 0.89; p = 0.006).

Children aged 0-59 months from families in the least poor or middle household wealth index categories had reduced odds of being stunted compared to those from the poorest families. Increased age of the child was statistically associated with stunting in children aged 0-59 months (AOR = 1.03, 95%CI: 1.02 - 1.04; p < 0.001). Girls aged 0-59 months had statistically significantly reduced odds of being stunted compared to boys aged 0-59 months (AOR = 0.72, 95% CI: 0.58 - 0.90; p = 0.005).

#### Risk factors for severe stunting

As shown in Table 3 the AOR indicated that children aged 0-23 months from families in the least poor or middle household wealth index categories had reduced odds of being severely stunted compared to those from the poorest families. Increasing age of the child was significantly associated with severe stunting (AOR = 1.08, 95%CI: 1.05 - 1.12; p < 0.001). Boys aged 0-23 months had increased odds of being severely stunted compared with girls aged 0-23 months (AOR = 0.58, 95%CI: 0.42 - 0.81; p = 0.002).

Children aged 0-59 months from least poor families had reduced odds of being severely stunted (AOR = 0.52, 95%CI: 0.33 - 0.83; p = 0.005) compared with those from middle and poorest families. Increasing age of the child was significantly associated with severe stunting in children aged 0-59 months (AOR = 1.02, 95%CI: 1.01 - 1.03; p < 0.001). Boys aged 0-59 months had increased odds of being severely stunted compared to girls aged 0-59 months (AOR = 0.72, 95%CI: 0.58 - 0.90; p = 0.005).

## Discussion

This paper presents data on the prevalence and risk factors associated with stunting and severe stunting in children in North Maluku province of Indonesia. This is the first study to assess the prevalence and factors associated with stunting and severe stunting in children aged less than five years in North Maluku province of Indonesia.

The prevalence of stunting in children in this population was high with 29% of the children aged 0-23 months and 38.4% of the children aged 0-59 months being stunted while, 14.1% and 18.4%, respectively were severely stunted. This level of stunting in North Maluku was higher than the national level among children aged 0-59 months of 28.6% reported in 2004 [20].

The prevalence of stunting and severe stunting was higher in children aged 24-59 months (50% vs 24%, respectively) than those children aged 0-23 months. This findings is similar to the results from Bangladesh, India and Pakistan [21-23] where children aged 24-59 months were found to be at a greater risk of being stunted. This suggests that for children aged 24-59 months stunting is not likely to be reversible [24]. Our findings support the previous assertions that the prevalence of stunting remains constant after 2 or 3 years of life [25].

Comparing the present study with children in four regions (Africa; Asia, Latin and South America), the prevalence of stunting in children 0-59 months in North Maluku province of Indonesia was slightly lower than that of Africa (40.1%), higher in Asia (31.3%), Latin America (16.1%) and South America (13.8%)[8]. These differences in prevalence likely result from a combination of factors like environmental, cultural differences and prolonged civil conflicts or war.

The results indicate that the gender of the child is a strong predictor of stunting and severe stunting in children aged 0-23 months and children aged 0-59 months. Girls had lower odds of becoming stunted or severely stunted compared to boys which supports the findings of other studies [26-28]. During infancy and childhood, girls were less likely to become stunted and severely stunted than boys and infant girls survive in greater numbers than infant boys in most developing countries including Indonesia [26-28].

Multivariate analyses identified that the following factors were statistically significantly associated with stunting and severe stunting for all of the three age categories after controlling for potential confounders: (a) number of family meals per day (2 meals per day); (b) child's age in months; (c) male sex; (d) household wealth (poorest); (e) parental employment (not working) and (f) district (Central and South Halmahera). These findings support similar studies indicating that mother's education, household wealth, gender, age and employment were significantly associated with stunted children [28-30].

This study suggests that stunting in children in North Maluku may be reduced by improving mother's education, mother's nutritional information and reducing poverty. This study highlights the need to provide special attention to reducing stunting in the Central and South Halmahera districts. Interventions for improving the provision of education for girls are required as lack of education appears to be a major risk factor for stunting in children in North Maluku province.

A number of important limitations due to the nature of the data used (secondary data analysis) needs to be considered. Firstly, there is no dietary intake data to support our findings. Secondly, this study used only two main characteristics (family and child level factors). Finally, the design is cross-sectional and reports only a "snapshot" of the frequency of stunting and severe stunting. Hence no strong conclusions can be made as to the possible causes of stunting and severe stunting.

Despite these limitations, the findings from this study contribute to our understanding of the factors associated with stunting and severe stunting in children aged 0-59 months in North Maluku, Indonesia. The findings in this study will assist the local government in North Maluku develop an appropriate intervention for the children aged 0-59 months and their parents. It may also help the Indonesian government public health planners develop a national nutrition program and interventions targeting young children especially in post conflict areas in eastern Indonesia. However, further research is required to understand the dietary and other determinants (including environmental risk factors) of stunting in North Maluku province of Indonesia.

## Conclusion

Childhood malnutrition remains a major public health problem in Indonesia. Results from this cross-sectional study showed that child's age in months, low socioeconomic status and gender (being a male child) were significant risk factors for stunting and severe stunting in North Maluku province of Indonesia. These results highlight the need for early intervention programmes aimed at reducing undernutrition in children, especially in the first two years of life.

## Competing interests

The authors declare that they have no competing interests.

## Authors' contributions

R and MJD designed the study. R, KA and SJB carried out the statistical analysis. R and KA wrote the manuscript. All authors made contributions to the interpretation of results and revised the manuscript for important intellectual content. All authors read and approved the final version of the manuscript.

## Pre-publication history

The pre-publication history for this paper can be accessed here:


